# Detecting (non)parallel evolution in multidimensional spaces: angles, correlations and eigenanalysis

**DOI:** 10.1098/rsbl.2021.0638

**Published:** 2022-02-16

**Authors:** Junya Watanabe

**Affiliations:** Department of Earth Sciences, University of Cambridge, Downing Street, Cambridge CB2 3EQ, UK

**Keywords:** allometric space, directional statistics, high-dimensional data, parallel evolution, phenotypic trajectory analysis

## Abstract

Parallelism between evolutionary trajectories in a trait space is often seen as evidence for repeatability of phenotypic evolution, and angles between trajectories play a pivotal role in the analysis of parallelism. However, properties of angles in multidimensional spaces have not been widely appreciated by biologists. To remedy this situation, this study provides a brief overview on geometric and statistical aspects of angles in multidimensional spaces. Under the null hypothesis that trajectory vectors have no preferred directions (i.e. uniform distribution on hypersphere), the angle between two independent vectors is concentrated around the right angle, with a more pronounced peak in a higher-dimensional space. This probability distribution is closely related to *t*- and beta distributions, which can be used for testing the null hypothesis concerning a pair of trajectories. A recently proposed method with eigenanalysis of a vector correlation matrix can be connected to the test of no correlation or concentration of multiple vectors, for which simple test procedures are available in the statistical literature. Concentration of vectors can also be examined by tools of directional statistics such as the Rayleigh test. These frameworks provide biologists with baselines to make statistically justified inferences for (non)parallel evolution.

## 1. Introduction

Multivariate approaches have proven to be powerful means to analyse phenotypes, yielding more holistic and nuanced understanding of organismal evolution and development than achievable from univariate approaches. It is now fairly common to conceptualize and analyse patterns of phenotypic evolution in multidimensional trait spaces (e.g. [1–7]). However, increasing dimensionality sometimes poses challenges in interpreting and analysing quantities that superficially appear familiar. This review concerns technical aspects of the analysis of phenotypic trajectories in multidimensional spaces, with a particular focus on the angles and their applications to detection of parallel evolution. Here, the term parallel evolution is used in the geometric sense; parallelism between trajectories in a trait space between multiple ancestor–descendant pairs [7,8], which typically results in acquisition of similar derived traits in the descendants. Parallel responses to similar selection pressures between lineages are often regarded as evidence for repeatability or predictability of phenotypic evolution under natural selection, and the prevalence and extent of such parallelism are under active debate (e.g. [9–11]).

A variety of toolkit exists for analysing evolutionary or developmental trajectories in multidimensional spaces. One useful concept is the allometric space [12–16], where variation among multivariate allometric axes (typically principal component (PC) vectors; [17–21]) can be visualized and analysed in various ways by treating empirical allometric axes as observations [22–29]. Another broadly employed tool is the phenotypic trajectory analysis [3,30–32], which primarily concerns quantification and statistical testing of inter-population differences in the magnitude, direction and shape of phenotypic trajectories.

The phenotypic trajectory (or phenotypic change vector) analysis has recently fuelled investigations into the parallel evolution [7,33]. There is a trend to quantitatively analyse patterns of evolutionary changes in putatively parallel lineages (e.g. [34–36]). The angles between phenotypic change vectors of different lineages play an especially pivotal role in empirical analyses of parallel evolution (e.g. [33,37–43]), because they are supposed to provide ‘intuitive and mathematically formal’ measures of (non)parallelism [37, p. 6].

**Figure 1.**
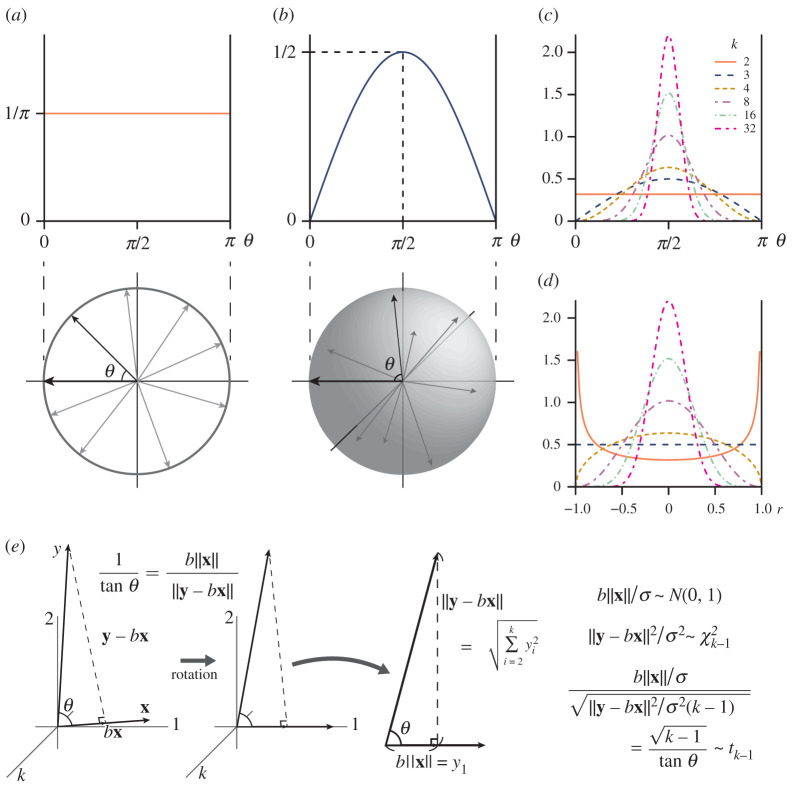
Distribution of angle in multidimensional spaces. (*a*,*b*) Probability density of angle *θ* between two vectors uniformly distributed on two-dimensional circle and three-dimensional sphere, respectively. Lower panels show schematic illustrations of the angle between a ‘pole’ (the thick arrow pointing the left-hand side) and another vector uniformly distributed on the unit circle/sphere. Upper panels show the corresponding densities. (*c*) Density of *θ* in general *k*-dimensional cases (2.9). (*d*) Density of *r* = cos*θ* (2.8). (*e*) Scheme to find probability distribution of random angle in *k*-dimensional space (only the 1st, 2nd and *k*th coordinate axes are shown for obvious visual restrictions). See text for details.

Unfortunately, however, interpretation of angles in multidimensional spaces is not so straightforward. Consider, for example, the angle between randomly directed vectors in two- and three-dimensional spaces. It is convenient to fix one of them pointed at a ‘pole’ and to let the other be uniformly distributed on the unit circle/sphere (figure 1*a*,*b*). The probability density of the angle between these vectors is then proportional to the arc length and surface area for a given infinitesimal increment of ‘latitude’. One will notice that the density for the two-dimensional space is uniform (figure 1*a*), whereas that for the three-dimensional space is peaked at the ‘equator’ because this region encompasses more area per latitude than ‘polar’ regions (figure 1*b*). This simple example demonstrates that distributions of random angles depend on the dimensionality, warning against extending our intuition into high-dimensional spaces. Regrettably, few recent analyses of evolutionary parallelism have taken this trend into account. Frameworks to make statistically justified inferences on angles have essentially been lacking in the current empirical literature.

This paper gives a brief overview of methods to analyse angles in multidimensional spaces. Specifically, it first derives the probability distribution of the angle between random vectors under the null hypothesis that the vectors have no preferred directions. It is by no means novel to science or even to the biological literature, where relevant results have been used in one form or another (e.g. [44–47]). The primary aim here is to disseminate well-known results with theoretical underpinnings. Recently, a framework for analysing multiple vectors simultaneously via eigenanalysis of a vector correlation matrix was proposed [48], but this framework lacked clear justifications as to which summary statistic should be looked at. This study also gives an alternative interpretation and a simple test statistic for that framework regarding the same null hypothesis.

## 2. Theory

### (a) Preliminaries

Let us first review the definition of the ordinary (Pearson product-moment) correlation coefficient, which has a close relationship with angles between random vectors. For the bivariate random observations of size *N*, (*x*_1_, *x*_2_, …, *x*_*N*_) and (*y*_1_, *y*_2_, …, *y*_*N*_), the correlation coefficient *r* is defined as

2.1 $$r=\frac{\sum_{i=1}^{N}\left(x_{i}-\bar{x})(y_{i}-\bar{y}\right)}{\sqrt{\sum_{i=1}^{N}{\left(x_{i}-\bar{x}\right)}^{2}\sum_{i=1}^{N}{\left(y_{i}-\bar{y}\right)}^{2}}},$$

where $\bar{x}$ and $\bar{y}$ are the sample means: $\bar{x}=\sum_{i=1}^{N}x_{i}/N,\bar{y}=\sum_{i=1}^{N}y_{i}/N$. By using the matrix notation $x={\left(x_{1}-\bar{x},x_{2}-\bar{x},\ldots ,x_{N}-\bar{x}\right)}^{T}$ and $y={\left(y_{1}-\bar{y},y_{2}-\bar{y},\ldots ,y_{N}-\bar{y}\right)}^{T}$, where the superscript T denotes transpose, we can rewrite (2.1) as

2.2 $$r=\frac{x^{T}y}{\left\|x\|\|y\right\|},$$

where the numerator is the inner product, and ‖⋅‖ denotes the vector norm or length ($\|x\|=\sqrt{x^{T}x}$). Recall the geometric definition of the inner product,

2.3 $$x^{T}y=\|x\|\|y\|\cos\theta ,$$

where *θ* is the angle formed by **x** and **y** in their *N*-dimensional space. Then, we have

2.4 r=cos⁡θ.

That is, the correlation coefficient and the angle between random vectors are directly related through the cosine/arccosine transformation. Here, the range of *θ* is taken as [0, *π*] (in radians) so that a one-to-one, though negative, relationship exists between *r* and *θ*: in the case of perfect positive correlation, *r* = 1, the two vectors point to the same direction, *θ* = 0; in the case of no correlation, *r* = 0, the two vectors are perpendicular to each other, *θ* = *π*/2.

We could standardize the variables by their standard deviations beforehand: $u=\|x{\|}^{-1}x$ and $v=\|y{\|}^{-1}y$, so that

2.5 $$r=\cos\theta =u^{T}v.$$

Since ‖u‖=‖v‖=1, **u** and **v** denote points on the unit hypersphere in the *N*-dimensional space.

Technically, the sample-mean-centred vectors **x** and **y** are in an (*N* − 1)-dimensional space, because centring with the sample mean reduces the effective dimensionality—the so-called degree of freedom—of the original *N*-vectors by one. For normal (and other) variables, the distribution of *r* with *N* sample-mean-centred observations from a population with arbitrary mean is the same as that with *N* − 1 observations centred at a known population mean (e.g. [49,50]). For what follows, it is convenient to consider the latter with the population mean 0.

This discussion concerns the equivalence between correlations in the variable (trait) space and angles in the object (lineage, individual, etc.) space, but the same relationship also holds when the space labels are swapped, i.e. the equivalence between correlations in a lineage space and angles in a trait space. We now turn to the distribution of random angles with a general *k*-dimensional space.

### (b) Distribution of random angles

Let us consider a pair of random vectors **x** = (*x*_1_, *x*_2_, …, *x*_*k*_)^T^ and **y** = (*y*_1_, *y*_2_, …, *y*_*k*_)^T^ and the angle *θ* between them. The elements are assumed to be independently and identically distributed. Let *b* = (**y**^T^**x**)/(**x**^T^**x**), the ratio of the inner product between **x** and **y** to the squared norm of **x**. By the geometric definition of the inner product (2.3), the vector *b***x** points to the foot of the perpendicular from **y** to **x**, and the vector **y** − *b***x** denotes this perpendicular (figure 1*e*). In the terminology of regression, *b***x** and **y** − *b***x** are predictions and residuals, respectively, in the regression of **y** on **x** (without intercept). The angle *θ* is related to these vectors in the trigonometric relationship

2.6 $$\frac{1}{\tan\theta }=\frac{b\|x\|}{\left\|y-bx\right\|}.$$

The distribution of this quantity is heuristically derived here; see, e.g. [49–52] for formal proofs. Assume that the elements of **x** and **y** are normally distributed with mean 0 and variance *σ*^2^, and that these two vectors are independent. The standardized vectors $\|x{\|}^{-1}x$ and $\|y{\|}^{-1}y$ are uniformly distributed on the unit hypersphere in the *k*-dimensional space. We can rotate the coordinate axes arbitrarily as far as the distribution of *θ* is concerned; let $\|x{\|}^{-1}x={\left(1,0,\ldots ,0\right)}^{T}$ for simplicity. Then, the distribution of $b\|x\|=y^{T}(\|x{\|}^{-1}x)=y_{1}$ is normal with mean 0 and variance *σ*^2^. Also, that of $\|y-bx{\|}^{2}/\sigma^{2}=\sum_{i=2}^{k}y_{i}^{2}/\sigma^{2}$ is *χ*^2^ with *k* − 1 degrees of freedom, and independent of b‖x‖ (figure 1). Therefore, by the operational definition of the *t*-distribution—namely, the distribution of the ratio of a standard normal variate to the square root of a *χ*^2^ variate divided by its degrees of freedom, with the two variates independent from each other—the quantity

2.7 $$\frac{b\|x\|/\sigma }{\sqrt{\left\|y-bx{\|}^{2}/\sigma^{2}(k-1\right)}}=\frac{\sqrt{k-1}}{\tan\theta }=\sqrt{k-1}\frac{r}{\sqrt{1-r^{2}}}$$

has a *t*-distribution with *k* − 1 degrees of freedom. The probability density (or element) of *r* in this case can be derived by transforming that of the *t*-distribution:

2.8 $$\frac{1}{B(1/2,(k-1)/2)}{\left(1-r^{2}\right)}^{(k-3)/2}\;dr,\;-1\leq r\leq 1,$$

where *B*(*a*, *b*) is the beta function with the two parameters *a* and *b* (this is just a normalizing constant, whose value need not concern most readers) (figure 1*d*). Then the density for θ=arccos⁡r is, by noting |dr|=|−sin⁡θ dθ|,

2.9 $$\begin{array}{rl} & \frac{1}{B(1/2,(k-1)/2)}{\left(1-\cos^{2}\theta \right)}^{(k-3)/2}|-\sin\theta \;d\theta |\\ & =\frac{1}{B(1/2,(k-1)/2)}\sin^{k-2}\theta \;d\theta ,\;0\leq \theta \leq \pi . \end{array}$$

This density has a peak at *θ* = *π*/2, which is increasingly pronounced as *k* increases (figure 1*c*). Another useful expression can be derived for *s* = *r*^2^, by noting $\left|dr|=|ds/2\sqrt{s}\right|$ and duplication of the positive and negative branches for *r* in (2.8):

2.10 $$\frac{1}{B(1/2,(k-1)/2)}s^{-1/2}{\left(1-s\right)}^{(k-3)/2}\;ds,\;0\leq s\leq 1,$$

which is the density of the beta distribution with the parameters 1/2 and (*k* − 1)/2.

The same distribution can be obtained from looser conditions than assumed here. For example, **x** could be from any distribution as long as it is independent of **y** that in turn has a spherically contoured distribution [50,51]. Indeed, expressions equivalent to (2.9) and (2.10) can be obtained from purely geometric evaluation of the surface area of a hyperspherical cap [44,53], which is equivalent to the probability for a random vector uniformly distributed on the hypersphere to fall within the region (see also [46]). A similar geometric reasoning was in fact involved in Fisher’s [54,55] formal derivation of the *t*-distribution (see also [56, ch. 11]), so, to be strict, the above derivation was partly circular.

These results can be used for testing the null hypothesis that two phenotypic change vectors have no preferred directions (population means being (0, …, 0)^T^) and are independent from each other, by inserting the dimensionality of the trait space into *k*. In particular, the *p*-value for an observed angle can be calculated from the *t* statistic (2.7); example functions for the R environment [57] are provided in the electronic supplementary material. This is equivalent to the ordinary correlation test, where typically *k* = *N* − 1 (see above). When the polarities of the vectors are to be ignored (e.g. test for angles between eigenvectors), the beta distribution (2.10) can be used instead. An equivalent test is commonly used for testing differences between allometric axes (e.g. [45]).

### (c) Pairwise angles and correlations

The above results concern a pair of random vectors, which should suffice when there are only a few lineages to compare. When interest is in analysing a set of many lineages simultaneously (e.g. [37,40,41]), a convenient procedure is to construct a matrix of pairwise angles or correlations. Let **x**_*i*_ denote phenotypic change vectors of *p* traits from *n* lineages (*i* = 1, …, *n*), each starting from its respective ancestor, and arrange these in rows of the *n* × *p* matrix **X**. This matrix then is standardized so that each row has the length of unity:

2.11 $$Z=\mathrm{diag}(\|x_{i}{\|}^{-1})X,$$

where diag( · ) denotes an *n* × *n* diagonal matrix with the designated *i*th diagonal elements. Then we consider the following *n* × *n* inter-lineage correlation matrix

2.12 $$C=ZZ^{T}.$$

By construction, **C** is symmetric and its (*i*, *j*)th elements are the vector correlations between the *i*th and *j*th vectors (2.5), with the diagonal elements being 1. The rows need not be centred, and thus retain the full effective dimensionality of *p*, unless the traits themselves are linearly dependent (as is the case for shape variables; see below). Taking element-wise arccosines of **C** yields a matrix of pairwise angles. For the sake of discussion, let Γ be the population (true) correlation matrix corresponding to **C**.

It might be tempting to make statistical inferences by treating pairwise angles or correlations in these matrices as a sample: e.g. calculating mean and standard deviation from pairwise angles and conducting a test of locations, e.g. *t*-test, Wilcoxon rank-sum test. However, such inferences should be, if at all, made with caution, because pairwise angles and correlations are generally not independent from one another. Ordinary statistical tests assume the observations to be independent (or at least uncorrelated), and violation of this assumption leads to suboptimal performance, e.g. inflated type I error rates. Off-diagonal elements of **C** have non-zero covariances unless $\Gamma =I_{n}$, where **I**_*n*_ is the *n* × *n* identity matrix [58,59]. Similar should be the case for pairwise angles. Therefore, it is inadvisable to conduct tests for pairwise angles in this way, unless, perhaps, the covariances are appropriately taken into account (methods for which are available for correlations; [60,61]). Although a sensible Monte Carlo design could be constructed to accommodate the covariances, it is rather questionable whether tests on mean pairwise angles are of much practical use beyond testing the null hypothesis $\Gamma =I_{n}$. There are more straightforward ways to test this null hypothesis (below), and other cases hardly translate into particular values of mean pairwise angles.

### (d) Eigenanalysis and one-step test for multiple vectors

De Lisle & Bolnick [48] proposed to use eigenanalysis of the inter-lineage correlation matrix **C** to detect concentration of phenotypic change vectors in a trait space. That is, to consider spectral decomposition (or eigendecomposition) of **C**:

2.13 $$C=ULU^{T},$$

where **U** is an *n* × *n* matrix of eigenvectors, and **L** = diag(*l*_*i*_) is an *n* × *n* diagonal matrix of eigenvalues. Their motivation was to quantify the magnitude of parallelism and effective dimensionality of parallel trajectories in the trait space by analysing eigenvalues of **C**, which represent variances along the corresponding PCs. For those purposes, however, it is more straightforward to consider the *p* × *p* inter-trait cross-product matrix **A** and its eigendecomposition instead:

2.14 $$A=Z^{T}Z=VKV^{T},$$

where **V** is a *p* × *p* matrix of eigenvectors, and **K** = diag(*k*_*i*_) is a *p* × *p* diagonal matrix of eigenvalues. The non-zero eigenvalues of **C** and **A** are in fact identical (electronic supplementary material, appendix A). **C** provides a quick means to surmise closeness between phenotypic change vectors, as well as a useful test described below. However, concerning variation in the trait space, **V** and **K** are more interpretable than **U** and **L** because the former pair pertains to the *p*-dimensional trait space whereas the latter pertains to the *n*-dimensional lineage space (electronic suupplementary material, appendix A). The rest of this section addresses quantification and test of the magnitude of parallelism—the first objective of the eigenanalysis as originally proposed [48]. Brief comments on the second objective—determination of dimensionality of parallel trajectories—are given in appendix A.

One complexity in dealing with eigenvalues of these matrices is the presence of sampling error and bias, which render sample eigenvalues inaccurate estimators of the corresponding population eigenvalues (e.g. [62,63]). Regarding the null hypothesis test of no parallelism, it has been suggested to compare eigenvalues of **C** with Monte Carlo distributions of eigenvalues of matrices drawn from a Wishart distribution [48]. To be clear, that distribution pertains to unscaled cross-product matrices, so the generated random matrices should be scaled as correlation matrices (this scaling was not clearly mentioned in DeLisle & Bolnick's descriptions, although was involved in their computer codes). Although this procedure is potentially valid, it has not been clearly indicated which test statistic should be looked at in testing this null hypothesis.

Here, it is proposed that dispersion of eigenvalues in these matrices, or equivalently sum of squared correlation coefficients from **C**, can be an appropriate test statistic. Eigenvalue dispersion has been used to quantify covariation between traits [64–67], and its sampling properties are relatively well known [68]. Intuitively, if phenotypic change vectors are uniformly distributed in the trait space, eigenvalues of **A** (or **C**) exhibit low dispersion. If the vectors are concentrated in a single or a few directions, then the eigenvalues are highly dispersed.

In particular, it is possible to show the following equality regarding dispersions of eigenvalues of **C** and **A** (denoted *l*_*i*_ and *k*_*i*_, respectively) and sum of squared correlations (see appendix A):

2.15 $$\sum_{i=1}^{n}{\left(l_{i}-\bar{l}\right)}^{2}=\sum_{i=1}^{\;p}{\left(k_{i}-\bar{k}\right)}^{2}+\frac{n^{2}}{p}-n=2\sum_{i<j}^{n}r_{ij}^{2},$$

where $\bar{l}$ and $\bar{k}$ are the averages of eigenvalues, and *r*_*ij*_ are the (*i*, *j*)th elements of **C**. Under the null hypothesis that all vectors are independently directed from one another without preferred directions, the population (true) correlation coefficients are zero, or $\Gamma =I_{n}$. For *n* lineages, we take it as if *p* traits are observations. Under the multivariate normality of the elements of **X**, each of $r_{ij}^{2}$ is distributed as Beta[1/2, (*p* − 1)/2] (2.10) and hence has the mean 1/*p* and variance 2(*p* − 1)/*p*^2^(*p* + 2). Furthermore, it is possible to show that $r_{ij}^{2}$’s are uncorrelated with one another under the null hypothesis [68,69]. Therefore, the expectation and variance of the sum of squared correlations are:

2.16 $$\text{E}\left(\sum_{i<j}^{n}r_{ij}^{2}\right)=\frac{n(n-1)}{2p}\text{ and Var}\left(\sum_{i\text{<}j}^{n}r_{ij}^{2}\right)=\frac{n(n-1)(p-1)}{\;p^{2}(p+2)}.$$

From these moments, Schott [69] proposed the following high-dimensional asymptotic test for the hypothesis $\Gamma =I_{n}$. Under the condition *n* → ∞, *p* → ∞, and *n*/*p* → *γ* ∈ (0, ∞), the distribution of $\sum_{i<j}^{n}r_{ij}^{2}-n(n-1)/2p$ converges to the normal distribution with mean 0 and variance $\lim(\mathrm{Var}(\sum_{i<j}^{n}r_{ij}^{2}))=\gamma^{2}$. (Note that this condition is just a modest generalization from the ordinary large-sample asymptotic condition, *n* → ∞ and *p*/*n* → 0, which is equally unrealistic.) Empirical values of $\sum_{i<j}^{n}r_{ij}^{2}$ can be compared with the normal distribution with the above mean and variance (2.16), and a large deviation can be seen as evidence against the null hypothesis, suggesting concentration of vectors. Schott [69] showed by simulations that this test has a reasonable type I error rate (although slightly too liberal when *p* or *n* is small, e.g. less than 16, in which case Monte Carlo simulations can be used) and a power usually superior to that of the conventional likelihood-ratio test.

A caveat on this procedure is that the test statistic does not convey information on the signs of correlation coefficients. Therefore, this test does not distinguish unimodal and antipodal concentration patterns (neither do tests entirely based on eigenvalues). It is strongly recommended to inspect **C** or PC scores to surmise what type of deviation from the null is present (see below). If the detection of parallel signal is of specific interest, it is probably more adequate to use the Rayleigh test from the directional statistics (electronic supplementary material, appendix B).

## 3. Recommendations

Although the statistical toolkits described above enable tests of particular null hypotheses, it is strongly recommended to conduct exploratory analyses before those tests are applied, in order to surmise overall patterns in the data. A common option is to visualize metric relationships between ancestral and descendant states via an ordination method like principal component analysis (PCA). Complementary to this approach is to make ordination of phenotypic change vectors, as is done for allometric axes [12,13]. The latter can be obtained from the eigenanalysis of **A** (2.14). It would also be useful to visualize relationships between ordination axes and traits via biplot, or to explore potential structures with clustering approaches (see electronic supplementary material, appendix C).

The Schott and Rayleigh tests share the same null hypothesis that the directional vectors are uniformly distributed, but have different alternative hypotheses. The Rayleigh test is powerful in detecting unimodal concentration of the vectors, but will be senseless if the vectors show antipodal or girdle-like distributions. The Schott test can detect these forms of deviation from uniformity, but does not distinguish antipodal and unimodal patterns by itself. Choice between these different tests should be made according to their properties and biological/statistical hypotheses of interest.

## 4. Example analysis

Stuart *et al.*’s [37] dataset of lake–stream divergence in the threespine stickleback (*Gasterosteus aculeatus*) is re-analysed here for demonstration. The original data were pre-processed as described in electronic supplementary material, appendix D. The resultant dataset consists of 13 phenotypic change vectors in 80 nominal morphological traits: 41 linear measurements, 38 Procrustes-aligned shape coordinates and one centroid size (from two-dimensional geometric morphometric analysis of 19 full landmarks). The effective dimensionality of the vectors is 80 − 4 = 76, as 4 degrees of freedom are lost by Procrustes alignment (assuming that the configurations were projected onto the tangent space).

**Figure 2.**
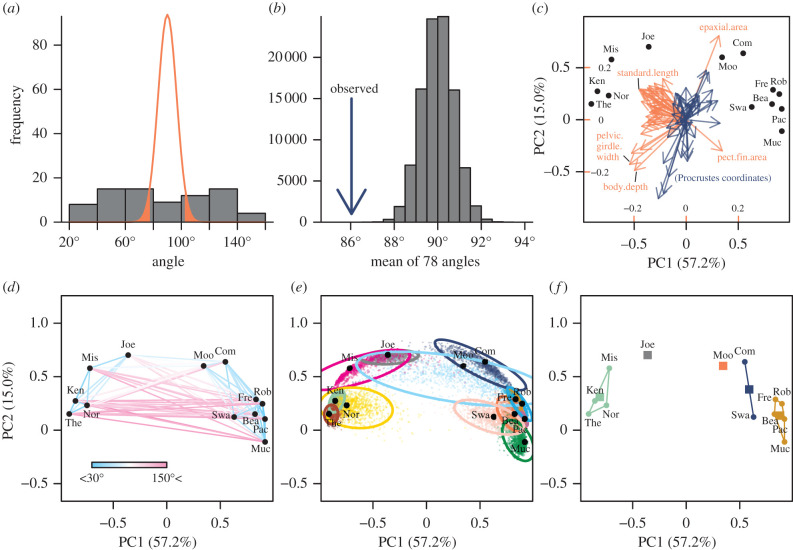
Re-analysis of Stuart *et al.*’s [37] dataset. (*a*) Histogram of 78 pairwise angles between the phenotypic change vectors in 13 lineages of *Gasterosteus aculeatus*, compared with a scaled density of random angles for *p* = 76 (2.9). Regions outside the 2.5 and 97.5 percentiles of the density are shown with solid orange fills. (*b*) Mean of the 78 pairwise angles (blue arrow) compared with the null distribution (histogram) based on 100 000 Monte Carlo simulation runs. (*c*–*f*) PC plots of phenotypic change vectors with different visualizations. (*c*) PCA biplot showing scores (points) and coefficients (arrows) of PC1 and PC2. The scaling parameter *α* was set to 1 (see electronic supplementary material, appendix C). Blue arrows denote shape coordinates, which cannot be interpreted individually, whereas orange ones denote the other traits, some of which are labelled. The inner axis labels are for coefficients, whereas the outer ones are for scores. (*d*) Pairwise angles shown with colour-scaled segments. (*e*) Clouds of bootstrap replicates and approximate 95% confidence ellipses. Ellipses are based on 5000 replicates of PC scores, but only 1000 replicates are shown for visual clarity. (*f*) Grouping with *k*-means clustering shown with colours and convex hulls. This grouping gave the smallest within-group sum of squares for (arbitrarily chosen value of) *k* = 5. Squares denote group centroids. Acronyms for watersheds are as in [37].

The resultant 78 pairwise angles ranged from 0.49 to 2.62 (28.0° − 149.9°). Compared with the null distribution of angles in the 76-dimensional space (2.9), 38 and 29 out of these were closer to parallel and antiparallel, respectively, than expected from random directions by chance alone (two-sided test at *α* = 0.05; no error rate control is deemed necessary for this demonstrative analysis; figure 2*a*). The mean angle of 1.50 (86.0°) was closer to parallel than expected for a mean of 78 random angles (*p* < 1 × 10^−5^ based on a Monte Carlo simulation with 10^5^ iterations; figure 2*b*). This interpretation is in stark contrast with that of Stuart *et al.* [37], who regarded their mean of 81.1° with 84 traits as ‘nearly orthogonal’. Note, however, that this test is for illustrative purposes only, as the mean pairwise angle lacks a clear interpretability (see above).

Ordination from PCA of the standardized phenotypic change vectors **Z** is shown in figure 2*c*–*f* (see electronic supplementary material, appendix C for details). The vectors of some lineages appear closely clustered with one another, but distribution of PC scores across the origin indicates that not all lineages had similar divergence (figure 2*d*). Non-parametric bootstrapping suggests that differences between vectors are mostly larger than what would be expected from sampling error alone except in most similar pairs (figure 2*e*). Nevertheless, the magnitude of sampling error appears heterogeneous among lineages, cautioning against face-value interpretation of differences; for example, sampling error in direction is evidently large for the Moore watershed, and this seems largely owing to small trajectory length. Potential clustering was explored with *k*-means clustering with varying numbers of clusters, and the result for *k* = 5 is shown as an example (figure 2*f*). PCA biplot shows that major components of variation among trajectories are to some extent characterized by variation in standard length and other traits highly correlated with it, along with several others (figure 2*c*). Overall, these exploratory analyses suggest the presence of multiple preferred directions of phenotypic change vectors.

In order to show deviation from the uniformity, the Schott test was applied to this dataset. The sum of the 78 squared correlations was 24.25, whereas the null expectation and standard deviation (from (2.16) with *n* = 13 and *p* = 76) were 1.03 and 0.16, respectively, indicating a statistically significant deviation from the null hypothesis of uniformity (*Z* = 144.08; *p* < 1 × 10^−10^). This test and examinations of pairwise angles and PC scores altogether indicate that the phenotypic change vectors most likely have preferred directions in the trait space, and that some of the vectors are significantly more (dis)similar to one another than expected from uniform distribution by chance. This insight is in contrast to the original account [37], and is partly in line with the reanalysis in [48], reinforced with more rigorous statistics. These results provide objective justifications to explore potential biological causes of the perceived patterns. In particular, the presence of multiple clusters may potentially reflect differing evolvability or selection regimes among recognized clusters. Such possibilities would deserve a more inclusive approach as was originally undertaken by Stuart *et al.* [37], with the aid of the present methodology.

## 5. Discussion

Angles have been commonly used in quantitative analyses of parallel evolution, but their properties in multidimensional spaces have not attained due attention. As clarified by the above analysis, angles between randomly directed vectors are peaked around the right angle in multidimensional spaces ((2.9); figure 1). It is therefore inadvisable to interpret angles at face value, e.g. angles closer to 90° than 0° regarded as evidence against parallel evolution on their own [33,37]. In addition, the dependency of the peakedness on dimensionality ((2.9); figure 1*c*) renders angles incomparable across different dimensions. Thus, direct comparison of angles or pooled meta-analysis across varying dimensionalities [33,38,70] will be tenuous, unless dimensionality is sensibly taken into account. A potentially useful standardization in this respect is $\sqrt{k-2}(\pi /2-\theta )$, whose distribution under the null condition (2.9) converges to the standard normal distribution as *k* → ∞ [52]; when *k* is sufficiently large, this quantity could be used as an effect size against the null distribution.

This review has concentrated on the null hypothesis that vectors are independent and have no preferred directions, which is just one of many hypotheses of potential biological interest [7,48]. This is not to claim superior biological importance of this hypothesis over another, but rather to present it as a baseline for analysing multidimensional vectors. At the other extreme, the hypothesis of completely parallel vectors could be tested, if interest is in detecting deviation from parallelism [7]. It is, however, more difficult to define a unified procedure for testing this null hypothesis than it may seem. It should in principle be possible to extend the present parametric framework into any arbitrary population values of correlation (although the distributions are substantially more complex). However, a practical test procedure will need to incorporate sampling error, whose nature and magnitude would largely depend on individual study systems. This is partly because complete correlation in the population eliminates any room for sampling variation, thereby trivially yielding sample correlation coefficient exactly 1 or −1 with probability 1. (On the other hand, tests against no preferred directions described above are not seriously affected by sampling error, with which the uniform distribution on the hypersphere typically remains unaffected under the null hypothesis [71].) A more realistic option will be to adopt one of the resampling-based approaches [3,20,32,72], as is done in the phenotypic trajectory analysis. However, it should be remembered that a resampling-based test, although being nominally non-parametric, is usually not free from the assumption that the populations share the same form of distribution, potentially differing only in the quantity of interest [73,74]. Between-group heteroscedasticity, whose presence was also suggested in the present re-analysis (figure 2*e*), can possibly undermine adequacy of tests of this type. Robustness of resampling-based tests against such cases needs to be critically assessed.

Apart from hypothesis testing, exploratory methods could be more commonly used in the analysis of parallelism. It should be straightforward to apply concepts and techniques originally devised for the analysis of allometric space to phenotypic change vectors. Examples include quantification of allometric disparity [14,24], test for shared trajectories in subspaces [75–77], and simultaneous visualization with phylogeny [15]. Although not fully discussed here, clustering approaches may also be useful in detecting patterns in multiple phenotypic change vectors (figure 2*f*; electronic supplementary material, appendix C).

It may be worth emphasizing that all statistical techniques described here fundamentally pertain to patterns of observed data rather than biological causalities. It is widely recognized that parallel and convergent evolutionary patterns (or lack thereof) can arise from a number of disparate causes [7,9,78]. Associating statistical techniques to any of such possibilities is restrained here, as it can lead to conflation between patterns and causalities and/or biased interpretation of statistical results [2]. It should be remembered that inferences for biological causality typically require more inclusive approaches than analysis of evolutionary patterns alone [79,80].

A paramount assumption in almost any geometric analysis of trajectories in a trait space [2,3,7] is that vectors can be meaningfully compared across different regions of the trait space. For angles to be meaningful, the space needs to be Euclidean [81,82]. This is, for example, when all traits are measured in the same unit. If traits are in different units (e.g. linear measurements and mass), the magnitude of measured angles can vary depending on the arbitrary choice of units. Log-transformation or standardization by mean or standard deviation would make traits nominally dimensionless, but it is generally an open question whether they ensure interpretability of vectors and angles.

The concentration of random angles around the right angle is just one of the potentially counterintuitive properties of high-dimensional spaces. Other superficially well-known concepts, such as volumes, Euclidean distances, and shapes of cubes and hyperspheres, also show peculiar behaviours in high-dimensional spaces [83,84]. Biological interpretations of evolutionary trajectories in high-dimensional trait spaces should be underpinned by proper understanding of the relevant geometry and statistics. In this regard, the literature of directional statistics (e.g. [71,85,86]) may potentially provide useful directions for future methodological developments.

## Data accessibility

This paper does not present any new data, but involves a re-analysis of a published dataset [37]. The data were retrieved from http://web.corral.tacc.utexas.edu/Stuart_2017_NatureEE_Data_Code/ on 17 August 2021. Codes to reproduce the re-analysis are provided as electronic supplementary material.
